# Evaluation of eco-friendly copper oxide nanoparticles for anticancer activity and antibacterial effects against *Streptococcus mutans* using molecular docking

**DOI:** 10.1038/s41598-025-23947-3

**Published:** 2025-11-20

**Authors:** Salem S. Salem, Abdullah Yousef, Ehab S. Abd El Hamid, Sara Ibrahim, Rania Hamed Elbawab

**Affiliations:** 1 Department of Botany and Microbiology, Faculty of Science, Al-Azhar University, Nasr City, Cairo, 11884 Egypt; 2 Basic & Medical Sciences Department, Faculty of Dentistry, Al-Ryada University for Science and Technology, Sadat City, Egypt; 3 Faculty of Dentistry, Ain Shams University, Cairo, Egypt; 4 Medical Laboratory Department, Medical Administration, University of Sadat City, Sadat City, Egypt

**Keywords:** Green synthesis, Copper oxide nanoparticles, Biofilm formation, Anticancer, Microbiology, Medical research

## Abstract

Since copper oxide nanoparticles (CuO-NPs) have antibacterial qualities, they are extremely helpful in many disciplines, including medicine. Utilizing data from FTIR, zeta potential, DLS, EDX, SEM, and UV–vis, the characteristics of the generated CuO-NPs were examined. CuO-NPs were found to have a spherical form, a surface charge of − 32.5 mV, and a maximum absorbance at 260 nm. Utilizing various methods, the produced CuO-NPs were tested for their antibacterial and anticancer qualities. On agar plates, CuO-NPs made from Mentha spicata leaf extract show fatal activity against *Streptococcus mutans* ATCC 25175 at high doses (0.5 mg/mL) includes a 16 mm diameter inhibitory zone. For CuO-NPs, the MIC was found to be 0.25 mg/mL. Furthermore, A dosage of 0.0625 mg/mL CuO-NPs was effective against the biofilm formation of *S. mutans* ATCC 25175 without affecting the growth of planktonic cells. Based on the findings, the dihydroorotase synthase (DHPR) may be partially or fully responsible for the activity, with the primary interaction seen being the hydrophobic contact with the amino acid residues in the pocket’s active site. It was demonstrated that CuO-NPs reduced OECM-1 cancer cells by 95.8% at a dose of 62.5 μg/mL. It was found that CuO-NPs had an IC_50_ value of 227.3 μg/mL on these cells.

## Introduction

Nanotechnology exhibits properties distinct from their bulk counterparts due to their larger surface area, enhanced interface interactions, and unique quantum effects^1–3^. Nanoscale metals can exhibit unique physical and chemical properties thanks to these traits, which are very useful in engineering, biology, and medicine^4,5^. For example, nanoparticles have been demonstrated to suppress both Gram-positive and Gram-negative bacteria^6,7^.

Green nanotechnology offers an environmentally responsible method of creating nanoparticles by fusing green chemistry and nanoscience. The significance of green synthesis techniques for creating metal and metal oxide nanoparticles has been brought to light by recent developments in nanotechnology^8^. By eliminating the need for hazardous chemicals and unfavorable reaction conditions, plant-mediated synthesis provides a sustainable, economical, and environmentally beneficial method. Plant extracts have been used in recent studies to demonstrate the green production of Ag/CuO nanoparticles, which have encouraging antibacterial and antifungal properties^9^. The synthesis of inorganic nanoparticles has advanced recently, highlighting their promise for a variety of biomedical uses^10^. Among these, metal nanoparticles are of particular interest for antimicrobial use, given their ability to mitigate bacterial resistance. Copper nanoparticles have shown strong antibacterial efficacy, including activity against drug-resistant and hospital-acquired pathogens^11^.

Dental plaque and cavities in the oral cavity are caused by* Streptococcus mutans.* To improve adherence to tooth surfaces, encourage glucan synthesis via glycosyltransferases, and enable survival in a variety of nutritional circumstances, this organism creates biofilms embedded in an extracellular matrix^12,13^. By means of matrix reinforcement, gene control, and quorum sensing, biofilm formation also shields bacteria against antimicrobial agents^14^. Reactive oxygen species (ROS) are created when bacterial membranes are broken down, interfering with quorum sensing, releasing copper ions, and breaking down the extracellular matrix, copper-based nanoparticles have been investigated as promising antibiofilm agents against *S. mutans*. These actions ultimately result in biofilm destabilization and bacterial cell death^15,16^. It is crucial to comprehend these pathways in order to create innovative strategies for preventing *S. mutans*-related oral illnesses.

Nanotechnology offers novel approaches to treating cancer in addition to infectious disorders. While nanoparticles provide benefits in drug administration, tumor targeting, and less systemic toxicity, conventional treatments are frequently linked to serious adverse effects and pharmacokinetic issues^17^. Due to their established anticancer efficacy in a variety of cell lines, cost-effectiveness, and biocompatibility, there has been particular interest in copper oxide nanoparticles (CuO-NPs)^18–22^. Size, shape, and surface chemistry of particles are some of the parameters that mediate their cytotoxicity and affect cellular absorption and inhibitory concentration (IC_50_) values^20^.

One practical and effective technique for CuO-NPs biomedical application is plant-mediated synthesis. when taking these benefits into account. During the creation of nanoparticles, polyphenols, terpenoids, and flavonoids are abundant in *Mentha spicata* act as capping and reducing agents. These phytochemicals may contribute to extra biological functions in addition to facilitating green synthesis.

Thus, the goal of the current study was to create CuO-NPs using *Mentha spicata* leaf extract, describe their physicochemical characteristics, and assess their molecular docking interactions, anticancer potential, and antibacterial efficacy against *S. mutans*. As far as we are aware, this is the first comprehensive study that combines in silico docking of CuO-NPs made from *M. spicata* with green synthesis, antibacterial testing, and cytotoxic evaluation. The uniqueness of our work is emphasized by this integrated approach.

## Materials and methods

### Preparation of *Mentha spicata * leaves extract

The leaves of *Mentha spicata* were gathered from the Al-Azhar University Faculty of Science garden in Cairo, Egypt. The Department of Botany and Microbiology recognized the plant, and placed in the departmental herbarium. The process of making *Mentha spicata* leaf extract was meticulous. After carefully selecting fresh mint leaves, they were expertly cleaned using double-distilled water. The leaves were first dehydrated in a drying oven at 50 °C for an entire night, and then they were ground with a mortar and pestle. 150 ml of distilled water were used to macerate 10 g of powdered leaves. To extract the bioactive ingredients, this mixture was heated to 100 °C for two minutes, allowed to cool to room temperature, and then filtered through Whatman 1 filter paper. The extracted sample was kept at 4 °C to get it ready for further examination.

### Synthesis of CuO-NPs

Sigma-Aldrich provided the copper nitrate. To create a precursor solution for the production of CuO-NPs, 0.0169 g of copper nitrate [Cu(NO₃)_2_] were dissolved to a concentration of 1 mM in 100 ml of distilled water. Following an undisturbed incubation time at 20 °C, fifty milliliters of the 1 mM copper nitrate solution were dropwise mixed with five milliliters of *Mentha spicata* leaf extract. CuO-NPs began to form when the colorless aqueous copper nitrate solution changed to a dark green tint. The color of the solution turned dark green as the synthesis progressed, signifying that the CuO-NPs had matured.

### Evaluation of CuO-NPs properties

Evidence of CuO-NPs synthesis was obvious due to a change in color. To give a comprehensive description of these nanoparticles, a range of analytical techniques were applied. CuO-NPs optical properties were clarified by means of UV–Vis spectroscopy. Identification of the biomolecules in the *M. spicata* extract and investigation of the surface chemistry of the CuO-NPs were made possible by FTIR spectroscopy. Together, the zeta potential and dynamic light scattering experiments provided insight into the nanoparticles’ surface properties and colloidal stability. Additionally, X-ray diffraction (XRD) was used to confirm the crystalline structure of the biosynthesized CuO-NPs, and Scherrer’s equation was utilized to ascertain the crystallite dimensions:

$$D=\frac{K\lambda }{\beta cos \theta }$$

where θ is the Bragg angle, K = 0.9, λ = 1.5406 Å, and β is the full width at half maximum (FWHM) of the diffraction peak in radians. The ratio of the integrated area of crystalline peaks to the total diffracted area (crystalline + amorphous) was used to measure the degree of crystallinity (%). The following relationship was used to calculate the specific surface area (SSA):

$$SSA=\frac{6}{D x p}$$

based on CuO’s predicted density of 6.31 g/cm^3^. Using the following formula, porosity (%) was determined by comparing the nanoparticles’ experimental density (ρexp) with their theoretical density (ρtheo):

$$\% {\text{Porosity }} = \, [({\text{exp}}P/theoP) - {1}] \, \times { 1}00$$

To verify the crystalline phase of CuO, the acquired diffraction peaks were lastly compared with common reference patterns from the JCPDS database.

The EDX analysis was used to describe the elemental composition and chemical analysis of the green-synthesized CuO-NPs. Ultimately, SEM analysis facilitated the observation of the particle form and dispersion.

### Antibacterial activity

#### Test organisms

For testing the antimicrobial and antibiofilm of CuO-NPs against *Streptococcus mutans* ATCC 25175.

### Antibacterial assay

#### Agar well diffusion assay

The agar well diffusion method was used to assess antibacterial activity of CuO-NPs. Once the bacterial strains were adjusted to 0.5 McFarland standard (≈1.3 × 10^8^ CFU/mL), they were injected into Mueller–Hinton agar (MHA) plates. A sterile cork borer was used to drill wells that were 6.5 mm in diameter. Then, each well received 30 μL of CuO-NPs solution (0.5 mg/mL). Sterile distilled water was used as the negative control and hexitol (2.5 mg/mL) as the positive control in each antimicrobial test. To allow for diffusion, the plates were refrigerated at 8 °C for 2 h before being incubated at 37 °C for the whole night. The diameter of the inhibitory zones was used to measure antibacterial activity. Every experiment was carried out three times, and the average results were published^21^.

#### Minimum inhibitory concentration (MIC)

The MIC of CuO-NPs was determined using the microbroth dilution method and 96-well microtiter plates. After being grown in nutritive broth, the bacterial suspension of *S. mutans* ATCC 25175 was adjusted to 0.4 McFarland standard (≈1.2 × 10^8^ CFU/mL). Each well had a final capacity of 200 μL, which contained 180 μL of bacterial inoculum and 20 μL of CuO-NPs solution at different concentrations. Positive controls contained bacterial culture without CuO-NPs, and negative controls contained only broth with CuO-NPs but no bacterial inoculum. Bacterial growth was detected at 620 nm at 37 °C using a microplate reader (Tecan Elx800, Fitchburg, WI, USA). MIC was defined as the lowest concentration of CuO-NPs that completely inhibited detectable bacterial growth. Assays were performed in triplicate, and mean values were given for each^22^.

#### Anti-biofilm activity of CuO-NPs

The ability of CuO-NPs to inhibit the formation of biofilms was tested using the *S*. *mutans* ATCC 25175 strain. Because nanoparticles were introduced to the growth media at the time of inoculation, the cells were able to form biofilms. CuO-NPs were serially diluted twice using a 96-well microtiter plate (MTP) with Trypticase soy broth supplemented with 1% glucose (TSBGlc). The dilutions were below the MIC values, ranging from 0.125 to 0.016 mg/mL. The MTP was then filled with bacterial suspensions that had a final concentration of 5 × 10^5^ CFU/mL per well. The impact of CuO-NPs on the development of the *S. mutans* ATCC 25175 strain was assessed using a microplate reader (Tecan Elx800, Fitchburg, WI, USA) at an optical density of 620 nm following a 24h incubation period at 37 °C. Comparing the treated wells with the untreated controls allowed for the calculation of the amount of biofilm formation in the presence of CuO-NPs as well as the percentage decrease. The average of the replicates was used to describe the outcomes of each test, which was carried out three times^23^.

### Experimental section of molecular docking simulation

The molecular docking process for CuO-NPs was modeled using the Molecular Operating Environment (MOE) version MOE 2014.0901^24,25^. The protein data band was used to download the 3D structures of the target receptors dihydroorotase synthase (PDB: 2VEG) and quorum sensing regulator (PDB: 4JVI). Both receptors’ active sites were produced by the previously reported method using just one chain (chain A)^24^. Dihydroorotase synthase (PDB: 2VEG) and quorum sensing regulator (PDB: 4JVI) were docked using GBVI/WSAdG as Rescoring 1 and London dG as Rescoring 2, and triangle matcher placement. By choosing chain A and adhering to conventional protocol, the active site was produced. Forcefield was used for post-placement refinement as well. The docking attitude had the highest binding energy with a negative value. Additionally, the structure of the CuO-NPs was constructed using the same procedure as previously reported^24^.

### Anticancer activity

#### Cell culture

For this study, the OECM-1 cell line was selected from Science Way Company in Cairo, Egypt. The OECM-1 cells were grown in F-12K media (Invitrogen, Karlsruhe, Germany) supplemented with 1% penicillin/streptomycin and 10% heat-inactivated fetal bovine serum (FBS). OECM-1 cells were maintained in DMEM medium. The OECM-1 cells were cultivated at 37 °C with 5% CO_2._.

#### MTT antitumor assay

The cytotoxicity of CuO-NPs was assessed using the MTT test. The mitochondria in living cells transform the hydrophilic tetrazolium salt, which appears yellow in this experiment, into the hydrophobic purple pigment formazan. The quantity of surviving cells is indicated by the color’s intensity. The MTT experiment was conducted using the previously described methods. After being cultivated in every well of a 96-well microtiter plate on the first day, the cells were suspended in 100 μL of the suitable medium at a density of 1.5 × 10^4^ cells/mL and incubated for 24 h. After being diluted in 0.5% DMSO and filtered using 0.450 μm syringe filters, the formulations were injected to the wells in triplicate at the appropriate quantities the next day. CuO-NPs were tested for OECM-1 cells purchased from Science Way Co. in Egypt at doses ranging from 0.4 to 100 μg/mL. A full day was spent cultivating the cells at 37 °C with 5% CO_2_. After the old media were swapped out for new ones on the third day, the cells were incubated for four hours at 37°C with 5% CO_2_. After this, a routine MTT assay was performed on them. Each sample was dissolved in 100 μL of 1% HCl in iso-propanol, and the absorbance at 570 nm was measured using a microplate reader. Plotting an exponential viability curve against the substance’s concentration allowed for the calculation of the inhibitory concentration at 50% (IC_50_)^26^. Cell viability percentage is equal to (absorbance control/absorbance sample) × 100.

## Result and discussion

### Copper oxide nanoparticles: production and characterization

**Fig. 1:**
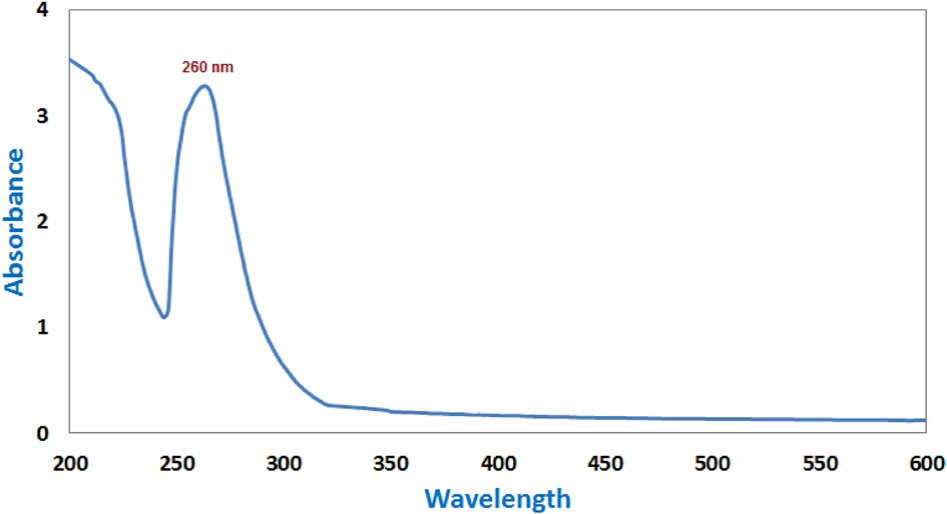
The UV–Vis spectrum for CuO-NPs produced by *M. spicata* leaf extract.

CuO-NPs were produced in this study using a plant extract (such as *M. spicata* leaf extract) and a green synthesis technique. The resulting dark green solution is a clear indication of metal reduction and CuO-NPs production. According to Phang et al.^27^, copper nanoparticles’ light-yellow to dark green hue is confirmed by UV-V absorption spectra, which are caused by the stimulation of surface plasmon oscillations within the particles. Verifying that CuO-NPs occur at 260 nm (Fig. 1). Incorporation In the as-prepared CuO-NPs produced in the presence of *M. spicata* leaf extract, surface plasmon bands (SPR) with a distinctive wavelength range of 260 nm are present, confirming the synthesis of CuO-NPs.

**Fig. 2:**
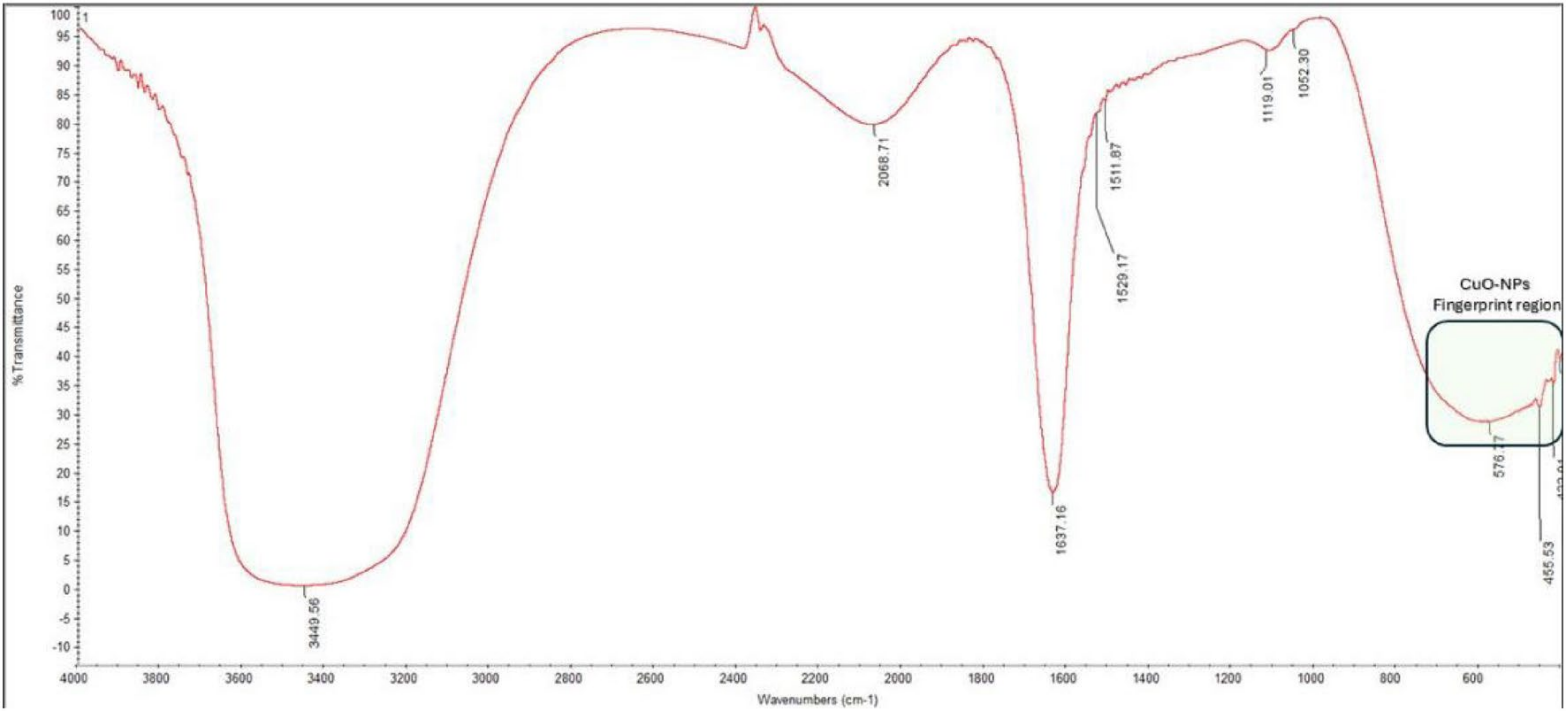
FT-IR spectra of the synthesized CuO-NPs.

Research using fourier to identify the biological molecules required for CuO-NPs capping, reduction, and stability, transform infrared [FTIR] spectroscopy is employed^28^. We looked at the band intensities of CuO-NPs in different locations. The FTIR spectra of the CuO-NPs displays several significant peak areas at 3409, 2913, 1637, 1382, 1051, and 595 cm^−1^. copper nanoparticles within the sample are functioning as a capping agent for the plant leaf extract, as suggested by spectra showing multiple minor shifts in peak positions. The signal at 595 cm^−1^ indicates the existence of CuO-NPs in the sample and an inorganic functional category. The peak at 1637 cm^−1^ can be attributed to either amide twisting or C=O stretching. The broad and strong peak at 3409 cm^−1^ is caused by the OH stretching vibrations of the extract’s carboxylic/phenol group, whereas the peak at 2913 cm^−1^ is caused by the phosphine stretch of the phytoconstituents. The C–N stretching showed the highest value at 1637 cm^−1^. The peaks at 1382 and 1051 cm^−1^ are believed to be caused by the CH group. The generated copper oxide nanoparticles’ FT-IR spectra show characteristic absorption bands in the 400–700 cm⁻^1^ area, which correspond to Cu–O stretching vibrations. This fingerprint site validates the formation of copper oxide nanoparticles, as seen in (Fig. 2). This demonstrates that functional groups have a major impact on the arrangement and creation of forms^29,30^.

**Fig. 3:**
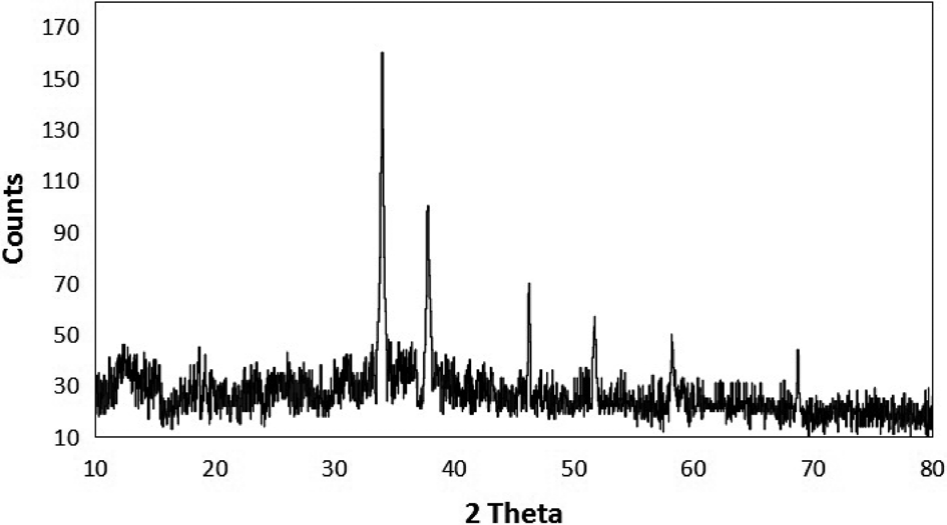
XRD pattern of CuO-NPs produced by *M. spicata* leaf extract.

Using an X-ray diffractometer, the crystalline character of the biologically produced CuO-NPs was verified in this investigation. Figure 3 displays the CuO-NPs XRD profile. The top points were indexed using the Powder-X program. At various angles—34°, 37°, 46°, 51°, 58°, and 68°. The miller indices for these peaks were, in order, 111, − 111, − 202, 020, and 022. The crystalline character of the biosynthesized CuO-NPs was confirmed by the XRD examination, which showed strong and crisp diffraction peaks^31^. Using Scherrer’s equation, the average grain size was determined to be between 17 and 28 nm. It is anticipated that the comparatively small crystallite size will increase the reactivity of the nanoparticles, potentially contributing to their strong antibacterial and anticancer properties. While the detected porosity (~ 18%) suggests the presence of structural voids that may further increase the surface reactivity, the estimated degree of crystallinity (~ 78%) indicates a highly organized structure. Since greater surface area-to-volume ratios offer more active sites for interaction with bacterial cell walls and cancer cell components, the computed specific surface area (22–36 m^2^/g) further supports the improved biological performance. Additionally, the creation of monoclinic CuO-NPs was confirmed by the obtained diffraction peaks matching the standard JCPDS card (No. 48-1548)^32^. CuO-NPs’ biological activity can be directly linked to their structural properties (small grain size, high crystallinity, moderate porosity, and large surface area), which supports the possibility of using them in antibacterial and anticancer treatments^33,34^.

**Fig. 4:**
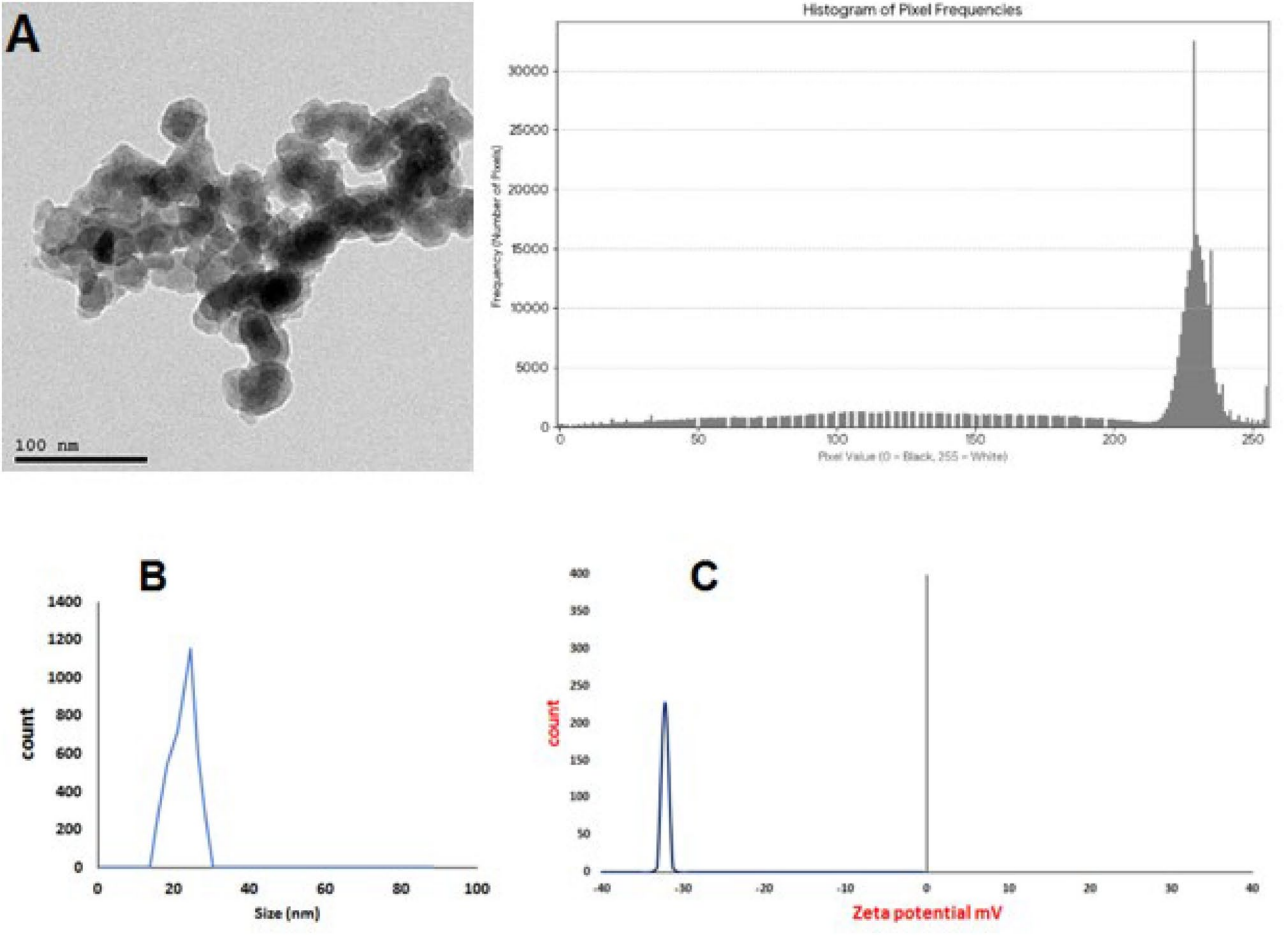
(**A**) TEM and PSA histogram, (**B**) DLS and (**C**) Zeta-potential measurements of CuO-NPs.

The size and morphology of the CuO-NPs generated by *M. spicata* leaves were examined using TEM. Most of the well-formed, spherical nanoparticles in the micrograph have sizes ranging from 10 to 47 nm. PSA Histogram representing a TEM image of CuO-NPs, demonstrates excellent image quality for quantitative analysis. The prominent peak at a high pixel value (approximately 230–240) corresponds to the bright background of the TEM image, while the broader distribution of lower pixel values represents the darker nanoparticles. Crucially, the absence of a peak at the maximum value of 255 indicates that the image is not overexposed and has no data clipping. This is vital for accurate analysis, as it confirms that all information in the brightest regions has been preserved, allowing for precise distinction between the nanoparticles and the background. The resulting image is ideal for accurate measurements of particle size and morphology, as seen in (Fig. 4A). Zeta potential techniques and dynamically scattered light (DLS) at various intervals were used to examine the surface and colloidal stability of as-prepared CuO-NPs (Fig. 4). The polydispersity index (PDI) of CuO-NPs was around 0.3, and the hydrodynamic diameter (HD) was approximately 21 nm, as can be shown in (Fig. 4B). They also had a zeta potential of − 32. 5 mV for CuO-NPs. The results (Fig. 4C) show that the CuO-NPs electrostatically attract the particles in the solution to one another. These results aligned with one previously reported by Yugandhar et al.^35^.

**Fig. 5:**
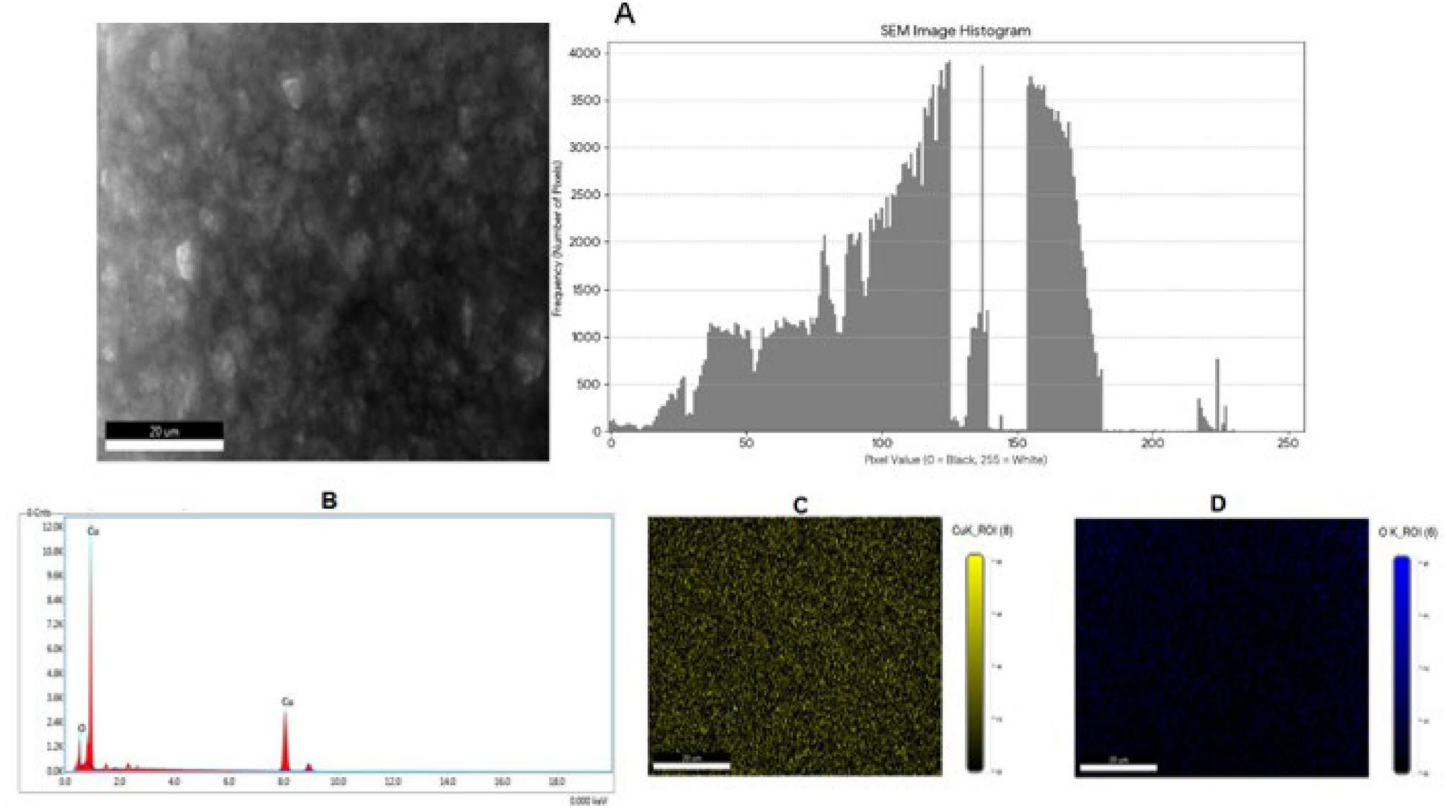
(**A**) SEM and PSA histogram, (**B**) EDX, mapping of Cupper (**C**) and Oxygen (**D**) of CuO-NPs produced by *M. spicata* leaves extract.

SEM, data mapping, and EDS analysis were employed in this study to verify the chemical makeup of CuO-NPs that were created biologically. The instrument was operated by continuous scanning in a wide range of angles. Figure 5A displays a SEM image of CuO-NPs, all of which have a sphere-like form. PSA histogram serves as a powerful visual representation of pixel distribution, revealing crucial insights into image quality. The histogram’s bimodal nature, characterized by two main peaks one wide, extended peak around a pixel value of 100 and a distinct second peak at 160 indicates that the image contains two primary components, such as the particles themselves and a different background or material phase. Critically, the absence of pixels at a value of 255 confirms that the image is not overexposed and no information has been lost in the brightest regions. This wide distribution of pixel values across a broad range signifies excellent contrast, making the image ideal for revealing intricate surface details and topography of the sample. Figure 5B illustrates the CuO-NPs EDX analysis. The EDS spectra and constituent image mapping confirm that Cu (yellow) and O (blue) are present in the CuO-NPs in a well-distributed form (Fig. 5C,D). These findings concurred with earlier research presented by Priya et al*.*^36^.

### Antibacterial and MIC of CuO-NPs against *S. mutans*

**Fig. 6:**
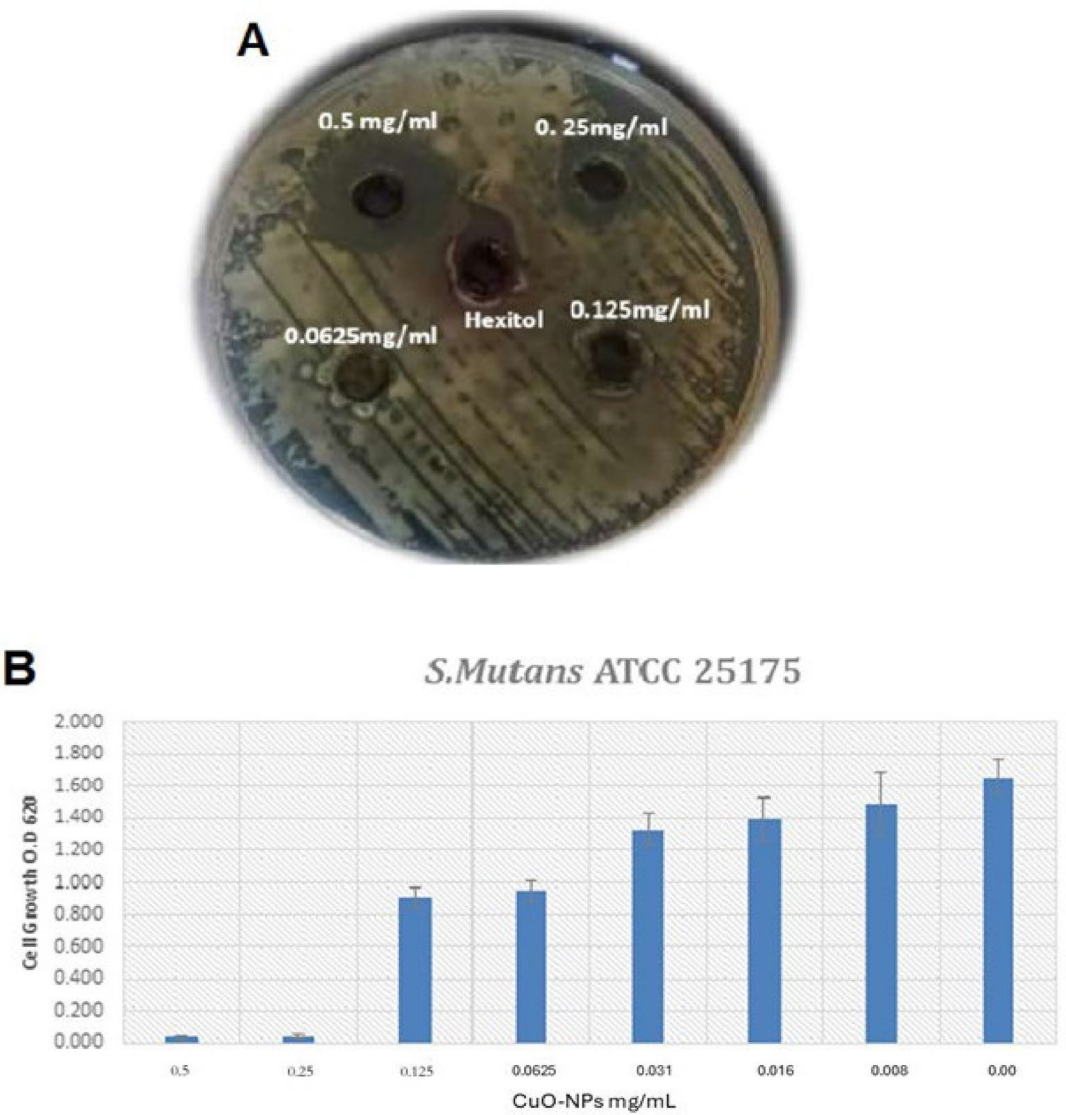
(**A**) Antibacterial and MIC of CuO-NPs against *S. mutans* compared with hexitol as positive control using agar well diffusion method. (**B**) Antibacterial and MIC of CuO-NPs against *S. mutans* using 96-well microplate.

The primary method by which CuO-NPs have antibacterial activity is by the generation of reactive oxygen species (ROS), which induce oxidative stress and damage bacterial cell membranes, proteins, and DNA. Furthermore, the permeability of the bacterial cell membrane may be increased by CuO-NPs interaction with it, and the intracellular proteins may be bound by the liberated Cu^2+^ ions, impairing vital biological functions^37^. These combined effects effectively restrict the development of germs, as demonstrated in our investigation against *S. mutans*. In the current study, it was demonstrated that* S. mutans *ATCC 25175 was susceptible to the potent antibacterial effects of CuO-NPs derived fro*m M. spicata* leaf extract. A lethal effect was observed at a high dose of 0.5 mg/mL, resulting in an inhibitory zone of 16 mm on agar plates. The MIC for CuO-NPs was found to be 0.25 mg/mL when compared to hexitol as a positive control, as shown in Fig. 6. The antibacterial qualities of plant-mediated CuO-NPs against oral infections have been assessed in several in vitro investigations^38^. It has been demonstrated that gram-positive and gram-negative bacterial strains are significantly reduced by CuO-NPs.  CuO-NPs have demonstrated bactericidal activity against periodontitis-causing microbes. Antibiotic-resistant bacterial and fungal infections can be effectively treated using medications that contain magnetic nanoparticles (NPs)^39^. Furthermore, biogenic CuO-NPs from gum Arabic showed antibacterial activity against *S. mutans*, with a MIC of 0.05 mg/mL and an inhibitory zone of 26 ± 1.0 mm^40^. When applied to *S. mutans*, the CuO-NPs made from *M. spicata* leaf extract showed antibacterial activity, which is consistent with previous studies reporting similar activity using other plant extracts such as *Morinda citrifolia* and *Carica papaya*. CuO-NPs made from *Morinda citrifolia*, for instance, CuO-NPs derived from *Carica papaya* exhibited more antibacterial properties than those found in commercial nanoparticles, while demonstrating antibacterial activity against *S. aureus* and *E. coli*^41^. *Nymphaea alba* Leaves extract was used to create a plant-mediated ZnO-CaO-CuO nanocomposite doped with Ag and Se in a recent work. This nanocomposite has shown remarkable biological activities and improved photocatalytic performance. The findings imply that copper-based nanomaterials’ stability, photocatalytic activity, and antibacterial qualities can all be enhanced by adding dual-doping components and employing green manufacturing techniques. This confirms the findings of our investigation, which showed that copper nanoparticles had encouraging photocatalytic and antibacterial properties^42^. Green synthesis can create nanoparticles with a controlled size (~ 21 nm), spherical morphology, and multifunctional properties like photocatalytic activity, anticancer effects, and ion-sensing capabilities, according to earlier research on plant-mediated nanocomposites, such as Se-doped ZnO-Al₂O₃ synthesized using *Nymphaea Pygmaea alba* extract. These results corroborate our study’s findings that CuO-NPs made using environmentally friendly techniques demonstrated strong antibacterial and photocatalytic activity, underscoring the potential of plant-mediated synthesis to create stable, bioactive, and multipurpose copper-based nanomaterials^43^.

### Effect of CuO-NPs on Biofilm activity of *S. mutans*

**Fig. 7:**
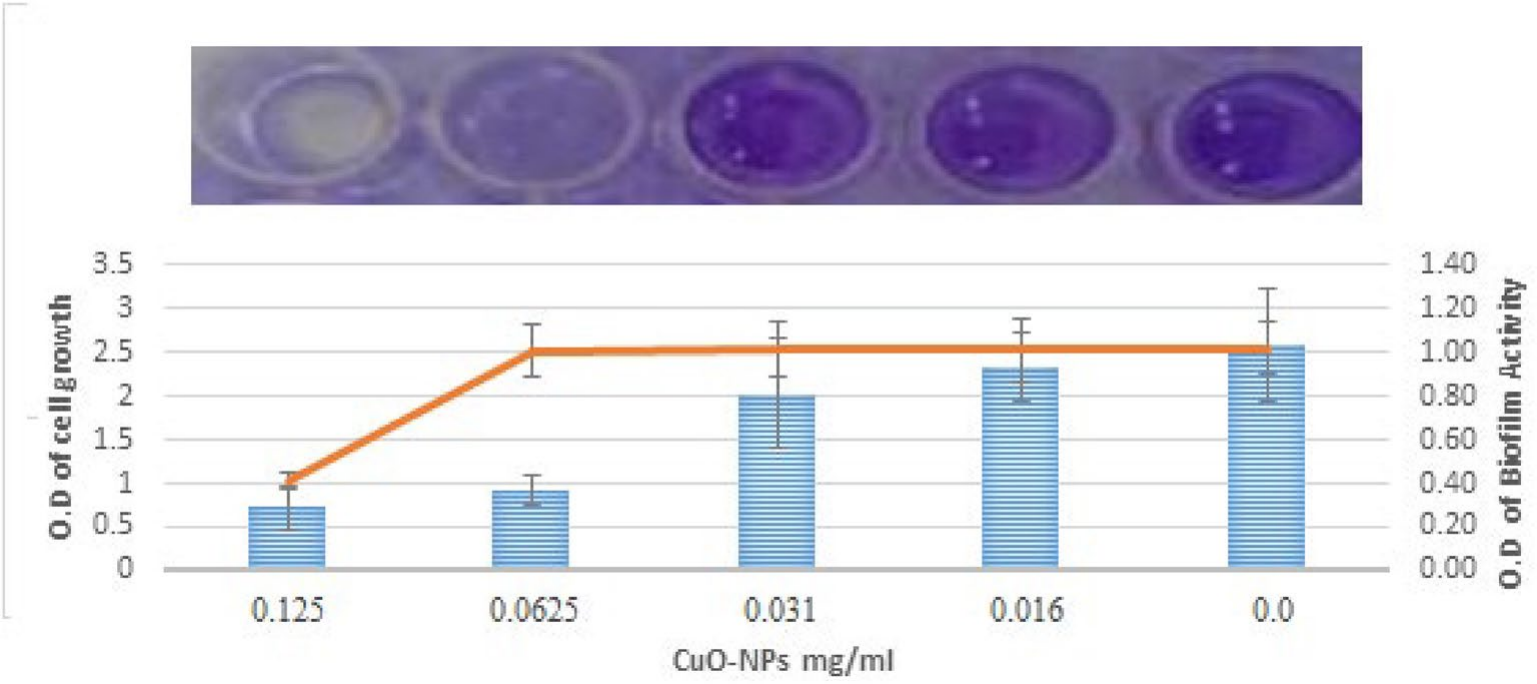
Effect of sub-inhibitory dose CuO-NPs on biofilm activity of* S. mutans*.

Without affecting the growth of planktonic culture cells, our study’s findings demonstrate that the CuO-NPs considerably reduce biofilm activity (p < 0.05) against *S. mutans* by 64.15 ± 0.7% at a dose of 0.0625 mg/mL as seen in Fig. 7. A dose of 0.016 mg/mLof TA-AuNPs totally eliminated produced *S. mutans* biofilm, according Selvaraj et al*.*^44^. The method by which metal and metal oxide nanoparticles inhibit infections is their capacity to modify the cell walls and membranes of resistant species, most likely due to the creation of pores on the cell wall and membrane^45^.

### Docking simulation

**Fig. 8:**
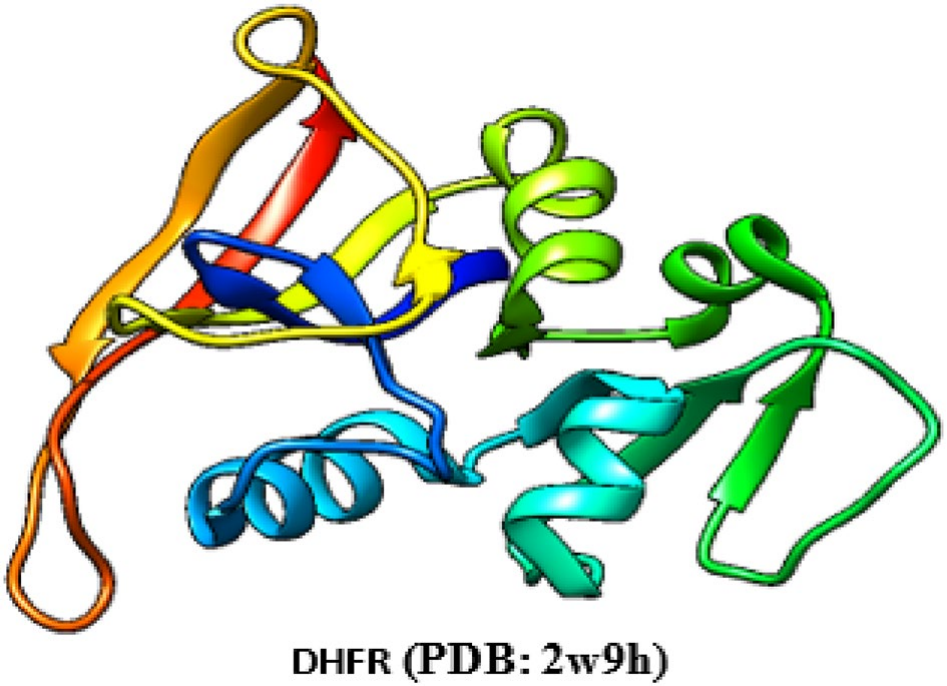
3D crystallography of the DHFR enzyme obtained from protein data bank.

The nanomaterial docking simulation can also reveal information about possible action pathways and response mechanisms^46,47^. For the CuO-NPs in the dihydroorotase synthase (DHFR) (PDB: 2w9h) active sites, docking simulation was performed in this work (Fig. 8). Finding out how CuO-NPs interacted with enzymes was the goal of the study to identify possible targets for antibacterial action. The dihydroorotase synthase (PDB: 2w9h) and the co-crystallized ligand were first measured for root mean square deviation (RMSD). The comparable values were 0.9216 Å and 0.7813 Å.

**Fig. 9:**
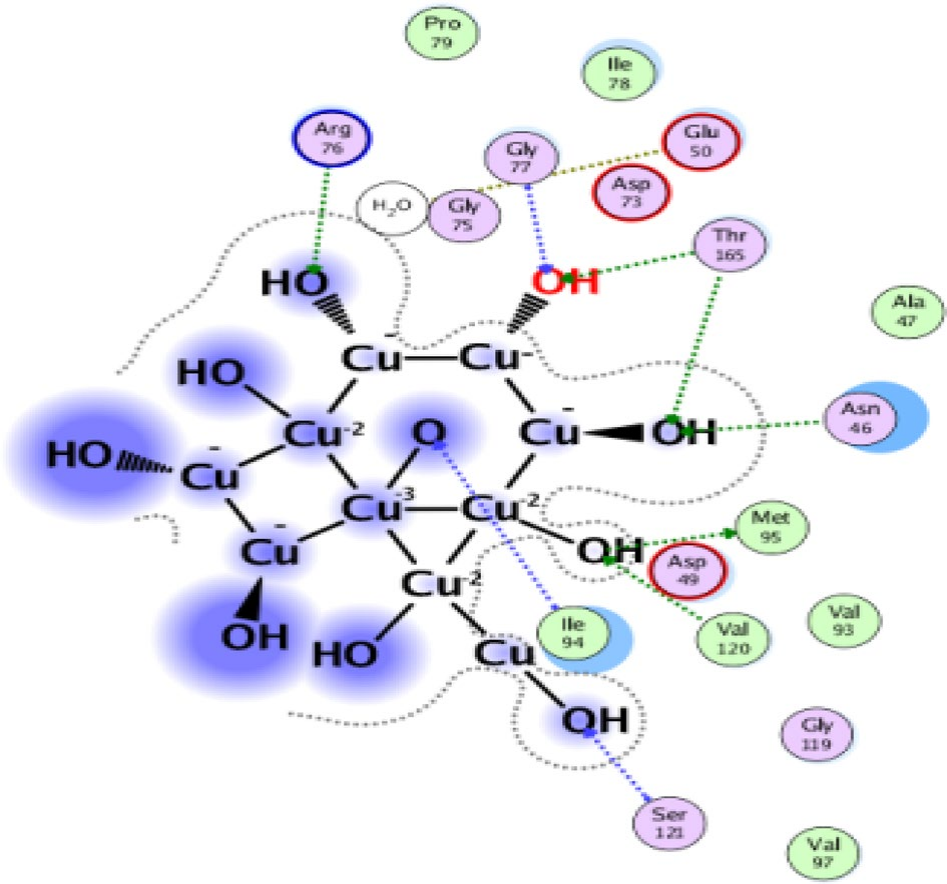
The binding mechanism of CuO-NPs inside the pocket with residues and amino acid interactions is demonstrated by the 2D structure of the CuO-NPs within the active site of dihydroorotase synthase (DHPR) (PDB: 2w9h).

**Fig. 10:**
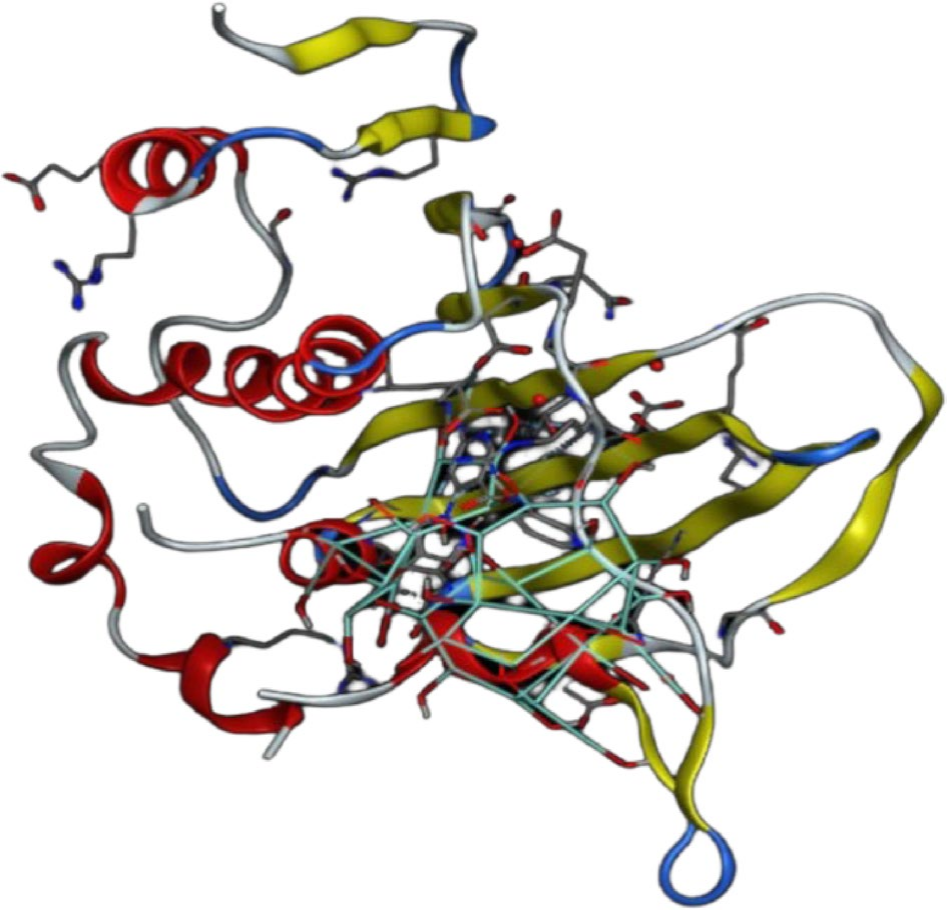
The binding mechanism of CuO-NPs inside the pocket with residues and amino acid interactions is demonstrated by the 3D structure of CuO-NPs within the active site of dihydroorotase synthase (DHPR) (PDB: 2w9h).

For the dihydroorotase synthase (DHPR) (PDB: 2W9h), CuO-NPs exhibited binding energy S =  − 3.65 kcal/mol and showed hydrophobic interaction inside the active site with many amino acid residues, such as Arg76, Gly77, Thr165, Asn46, Asp49,Ser171, Ile94, Val120, and Met95 (Figs. 9, 10). Lastly, we can say that CuO-NPs exhibit antibacterial activity by blocking the control of quorum sensing and dihydrorotase synthase. Nonetheless, the binding energy suggests that CuO-NPs prefer DHPS over quorum sensing control, and their activity is indicated by hydrophobic interactions in the active site pocket, which is smaller than 4.5 Å.

### CuO-NPs as antitumor agent using (MTT) assay

**Fig. 11:**
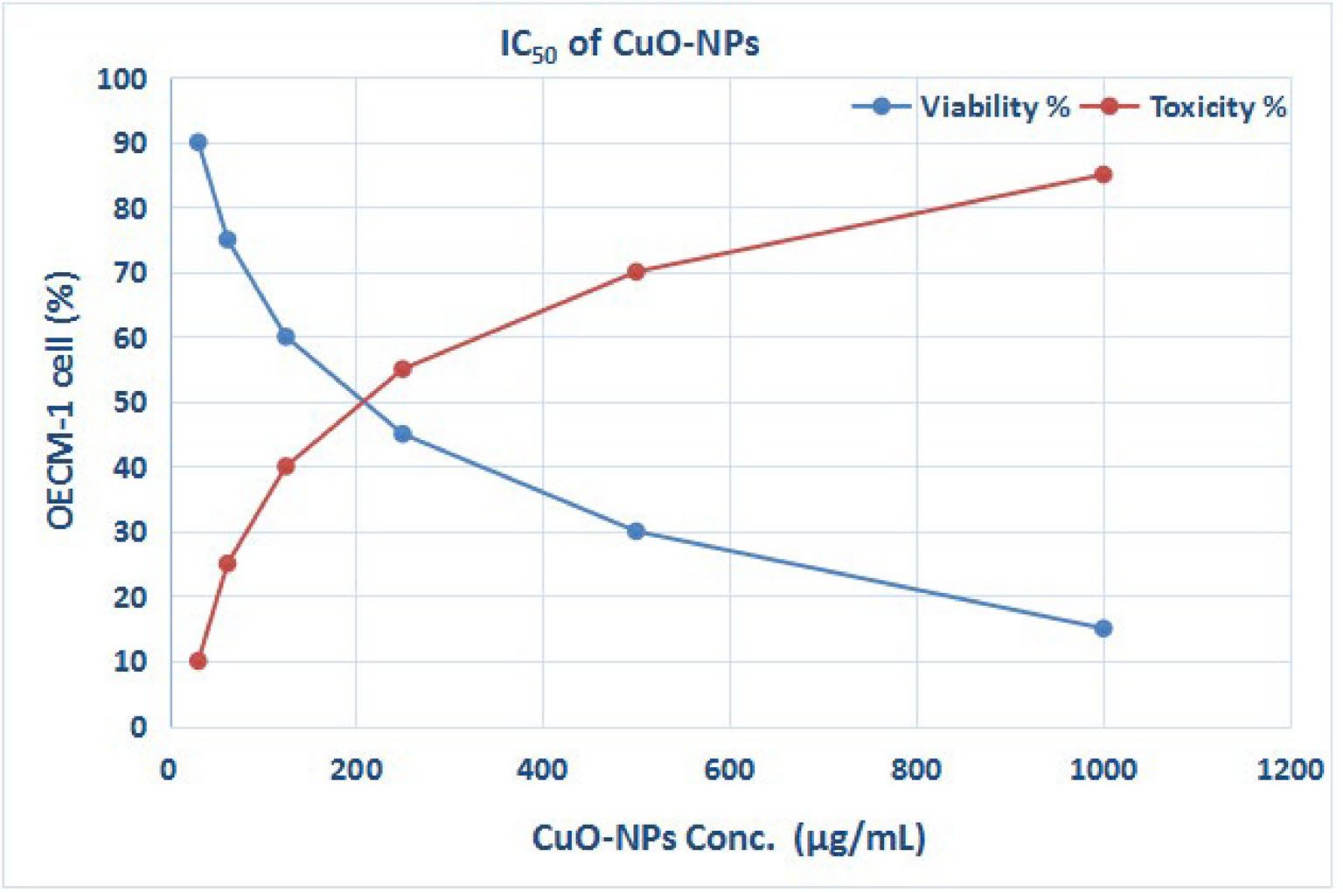
An interaction between CuO-NPs and OECM-1 cell line was conducted using MTT assay.

One of the most dangerous diseases in the modern world is still cancer. Every day, new ways to deal with it are being created. The use of MNPs has garnered a lot of attention lately because of its anticancer properties. The capacity to identify cancerous cells and deliver drugs precisely and precisely while avoiding interaction with healthy cells is one characteristic that can be added to nanoparticles^48,49^. It has been shown that environmentally friendly biocompatible nanoparticles have been developed. This action enables them to enhance their interaction with life systems more efficiently. Due to the numerous properties that nanoparticles possess, their harmful effects are significant. Several characteristics are encompassed in this category, such as surface chemistry, concentration, shape, and size^50,51^. According to a recent study, Ag/CuO nanoparticles produced utilizing environmentally friendly processes and plant extracts shown strong antibacterial and antifungal properties in addition to mild cytotoxic effects. These results demonstrate how well plant-mediated synthesis can increase the biological activity of nanoparticles, hence bolstering their potential uses in environmental and medicinal domains^52^. Figure 11 presents the findings of the antitumor analysis. According to the data in Fig. 11, the CuO-NPs showed an IC_50_ value of 227.3 μg/mL on OECM-1 cell lines. To evaluate the impact of CuO-NPs on OECM-1 cell lines, an anticancer study was conducted utilizing several dosages of CuO-NPs, ranging from 1000 to 31.25 μg/mL. The vitality of OECM-1 cells was 85%, 70%, 55%, 40%, 25%, and 10%, as illustrated in Fig. 11. Furthermore, the nanoparticles’ tiny size and surface charge improved their distribution and mobility inside the malignant matrix^53^. Subsequent investigation revealed that a nerve tumor cell line was significantly killed by CuO-NPs produced using *Boswellia thurifera* aqueous extract^54^. Copper nanoparticles also caused dose-dependent cytotoxicity in HepG2 and MCF-7 cells^55,56^. In another study, Caco-2 cells were exposed to copper nanoparticle dosages ranging from 31.25 to 1000 μg/mL. The anticancer activity showed an IC_50_ value of 96.28 μg/mL after a 48h exposure period^57^.

## Conclusion

To sum up, the application of enhanced CuO-NPs has shown great promise in managing bacteria that are resistant to many drugs, especially *Streptococcus mutans*. These nanoparticles’ effects on MIC and biofilm formation demonstrate their exceptional effectiveness in preventing bacterial growth. Ongoing research and development in this area may lead to the development of novel antimicrobial strategies to address the issue of multi-drug resistance in this dangerous bacterium. CuO-NPs docking simulation showed that the enzyme hybrid CuO-NPs complex was stable, and the DHFR might be a possible mode of action. The effect of CuO-NPs on OECM-1 cells, particularly their capacity to lower cell viability, was examined using the MTT method. For example, OECM-1 cells demonstrated a 95.8% reduction of cancer cell proliferation at a dose of 62.5 μg/mL. CuO-NPs were shown to have an IC_50_ value of 227.3 μg/mL on OECM-1 cancer cells. All things considered, the combination of CuO-NPs’ antibacterial and anticancer properties shows considerable promise as a biological agent.

## Data Availability

Upon reasonable request, the corresponding author will make the datasets created and/or examined during the current work available.
